# 4431 Utilization of swept source optical coherence tomography to optimize characterization of cystoid macular edema in preterm infants

**DOI:** 10.1017/cts.2020.320

**Published:** 2020-07-29

**Authors:** Kai Seely, Shwetha Mangalesh, Katrina Winter, Vincent Tai, Du Tran-Viet, Stephanie Chiu, Cynthia Toth

**Affiliations:** 1 Duke University

## Abstract

OBJECTIVES/GOALS: The goal of this study is to evaluate and optimize the characterization of cystoid macular edema (CME) using an investigational swept source (SS)-OCT system. Our knowledge of CME in preterm infants is limited; optimizing its characterization is a critical step in understanding its impact on vision. METHODS/STUDY POPULATION: In this IRB-approved protocol, 118 preterm infants were imaged in the Duke intensive care nursery (ICN) with a novel lightweight, hand-held, high-speed, SS-OCT system following routine clinical eye exams. SS-OCT images were deidentified, automatically segmented using custom software (DOCTRAP), measured for several retinal layer thicknesses, and reviewed by masked expert graders for the presence and severity of CME. Reliability of SS-OCT measures will be assessed, and the association between CME status and retinal layer thicknesses will be calculated using logistic regression modeling. RESULTS/ANTICIPATED RESULTS: The prevalence of CME overall and by severity will be calculated. The distribution of several retinal layer thicknesses will be reported and compared by infant CME status and, when edema is present, by CME severity. Reproducibility and repeatability will be reported for objective variables, and intra-grader and inter-grader agreement will be reported for subjective variables. Multivariate logistic regression coefficients and odds ratios will be calculated for each retinal layer thickness variable. DISCUSSION/SIGNIFICANCE OF IMPACT: This study will use a novel SS-OCT system to identify retinal thickness measures that may be objective markers of CME status. This will refine the characterization of CME and provide a framework for correlating CME with functional outcomes like visual acuity. CONFLICT OF INTEREST DESCRIPTION: SC and CT have unlicensed patents on relevant technologies. CT receives royalties from Alcon and Hemosonics and consultation fees from EMMES.

